# Study on Interaction Between TATA-Box Binding Protein (TBP), TATA-Box and Multiprotein Bridging Factor 1(MBF1) in *Beauveria bassiana* by Graphene-Based Electrochemical Biosensors

**DOI:** 10.3389/fchem.2020.00278

**Published:** 2020-04-15

**Authors:** Chi Song, Zhijia Peng, Xiaogang Lin, Haoyue Luo, Min Song, Lifeng Jin, Xiangyue Xiao, Hong Ji

**Affiliations:** ^1^Department of Life Science and Technology, Changshu Institute of Technology, Changshu, China; ^2^Key Laboratory of Optoelectronic Technology and Systems of Ministry of Education of China, Chongqing University, Chongqing, China

**Keywords:** *Beauveria bassiana*, TATA-box binding protein (TBP), multiprotein bridging factor 1(MBF1), graphene oxide, electrochemical impedance spectroscopy (EIS)

## Abstract

The regulation of transcription level is an important step in gene expression process. *Beauveria bassiana* is a broad-spectrum insecticidal fungi widely used in the biologic control of arthropod. The regulation of its transcription level is a multilevel complex process. Multiprotein bridging factor 1(MBF1) is a transcriptional co-activator that bridges sequence-specific activators and the TATA-box binding protein(TBP), Little is known about the interaction between MBF1, TBP, and TBP binding to DNA(TATA-sequences)in filamentous fungi of *Beauveria bassiana*, The binding of TBP to TATA-box and TBP to MBF1 was investigated via electrochemical biosensor. Graphene oxide has an electronic mobility that is unattainable for any metal, so it will be highly sensitive as a test electrode. Hence, we developed a simple, sensitive and specific sensor based on an TBP probe and graphene oxide that successfully detected the interaction of TBP and TATA-box or MBF1. From the electrochemical impedance spectroscopy (EIS), we find that the radius will increase when adding TATA-box or MBF1 buffer to the modified TBP protein electrode. When adding no TATA-box or no MBF1, the radius is relatively unchanged. The interaction between TBP and TATA-box or MBF1 was proved based on the results. These data confirmed the specificity of the interactions, (1) our developed graphene-based electrochemical biosensor can be used for monitoring the interaction between TBP and TATA-box or MBF1, (2) TBP can bind to TATA-box, (3) TBP can bind to MBF1, and (4) TBP mediates the interactions of MBF1 to DNA. Therefore, this work provided a label-free, low-cost and simple detection method for the complex process of eukaryotic gene transcription regulation.

## Introduction

Transcription factors are a major group of important protein factors in cell life activities. They are active in the nucleus and regulate the expression of genetic genes, thus affecting all aspects of life. Transcription is a complex process of synthesis of RNA catalyzed by RNA polymerase, in which DNA is used as template and ATP, CTP, GTP, and UTP are used as raw materials. The transcription of eukaryotic cells can be divided into three categories according to different RNA polymerases: (1) RNA polymerase I transcription rRNA; (2) RNA polymerase II transcription mRNA; (3) RNA polymerase III transcription tRNA and other small RNA.

The TATA-box binding protein (TBP) is a key protein in transcription initiation of eukaryotic cells. And it is a universal transcription factor with 30 kD. At the same time, it is required for transcription initiation of RNA polymerase and the only universal transcription factor that can specifically bind to DNA (Lee and Young, 2000). In most eukaryotes, the TBP protein binds to a conserved sequence called TATA-box at about 25 bp on the promoter (Kornberg, 2007). TBP is an important component of transcription factor IID (TFIID). It, together with other transcription factors, constitutes TATA binding protein-related factors involved in transcription initiation and regulation of transcription activity (Tang et al., 1996).

At present, studies have found that nearly 30 species of protein can interact with TBP to form TATA binding protein-related factors (Davidson, 2003). Binding of TBP to the transcription coactivator MBF1 (Takemaru et al., 1997) *in vivo* and *in vitro* has been reported in humans, arabidopsis, and yeasts (Mariotti et al., 2000; Brendel et al., 2002; Liu, 2003).

In this work, in order to study the binding of TBP with TATA-box or MBF1 in filamentous fungus, we attempted to establish a biosensor based on electrochemical impedance spectroscopy to carry out the study. Graphene or graphene oxide (GO) has carrier mobility inaccessible to any metal and as a test electrode that is high sensitive to changes in electrochemical parameters (Shu et al., 2018; Zeng et al., 2018). The unique capacity of graphene or graphene oxide (GO) in adsorbing biomolecules such as nucleic acids and proteins will make graphene biosensor more sensitive and target-specific than other detection methods, such as chromatography, colorimetry, and fluorescence analysis (Hu et al., 2011, 2012; Wang et al., 2011; Pei et al., 2012; Xing et al., 2012). In addition, Electrochemical Impedance spectrum (EIS) use a small amplitude sine wave voltage (or current) as the disturbance signal and make the response of the electrode system to produce the approximate linear relationship (Newman, 1989). It is a kind of measuring method of frequency domain, so it can get more dynamic information and electrode interface structure information than conventional Electrochemical methods (Newman, 1989). At present, most biosensors based on electrochemical impedance spectroscopy focus on the interaction between antibodies and antigens (Katz et al., 2004; Hou et al., 2014). The impedance change on the electrode surface was measured by the electrochemical system to reflect the binding effects of the antigens and antibodies (Hou et al., 2013). Based on the principle, we adopt a more intuitive Nyquist diagram to reflect the binding effect of TBP and TATA-box or MBF1 in filamentous fungus (Hu et al., 2011). The variation of the semicircle radius in the Nyquist diagram reflects the impedance change at the electrode interface, so as to verify the binding effect of TBP and TATA -box or MBF1 in filamentous fungus.

## Mechanism of Experiment

**Figure 1:**
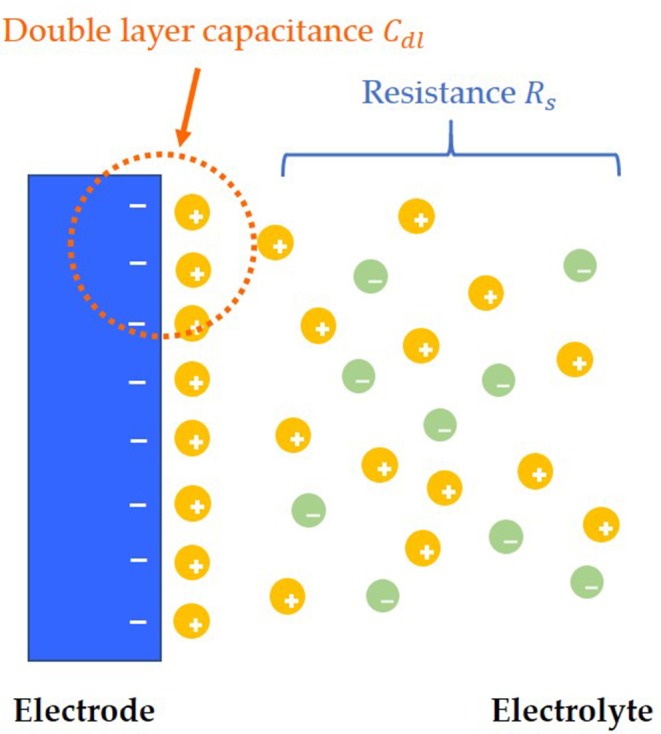
Electric double layers model.

After adding solution to the surface of the electrode, the charges near the interface between the electrode and the solution will be redistributed, and the opposite charges will be equally distributed on both sides of the interface, thus forming the simplest electric double layers model, which is also called the Helmholtz electric double layers model (Helmholtz, 1879; Christine et al., 2001). However, there is a flaw in the Helmholtz model, which assumes that the capacitance ***C***_***dl***_ of electric double layers is a constant value. However, in the experiment, ***C***_***dl***_ is a variable, which can be influenced by relative potential and the concentration of electrolyte. According to the concept of Helmholtz model, the improved model of electrode and solution interface distribution is shown in Figure 1 (Helmholtz, 1879).

**Figure 2:**
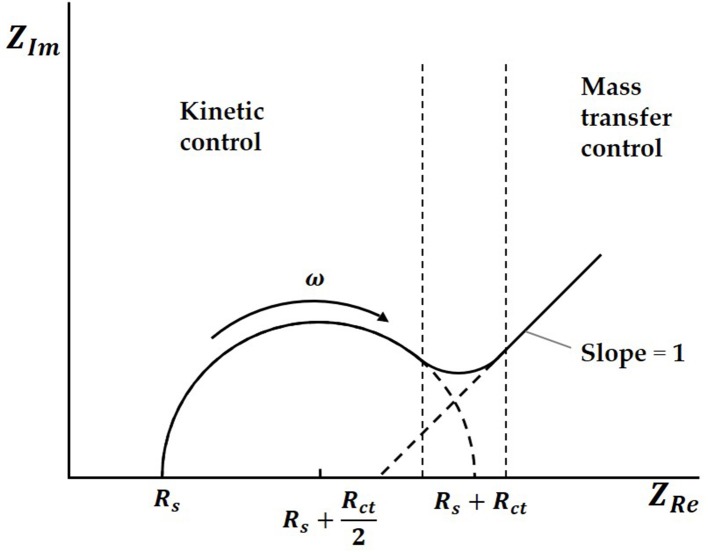
The typical EIS diagram.

Both charge transfer and diffusion control the process. The Nyquist plots are made up of a semicircle in the high frequency region and a straight line with a dip angle of 45° in the low frequency region. The high frequency area is controlled by charge transfer, while the low frequency area is controlled by diffusion of solution. In an ideal situation, the typical EIS diagram obtained is a curve with a semicircle and tail, as shown in Figure 2 (Prodromidis, 2010; Lu et al., 2011; Asadi et al., 2016).

**Figure 3:**
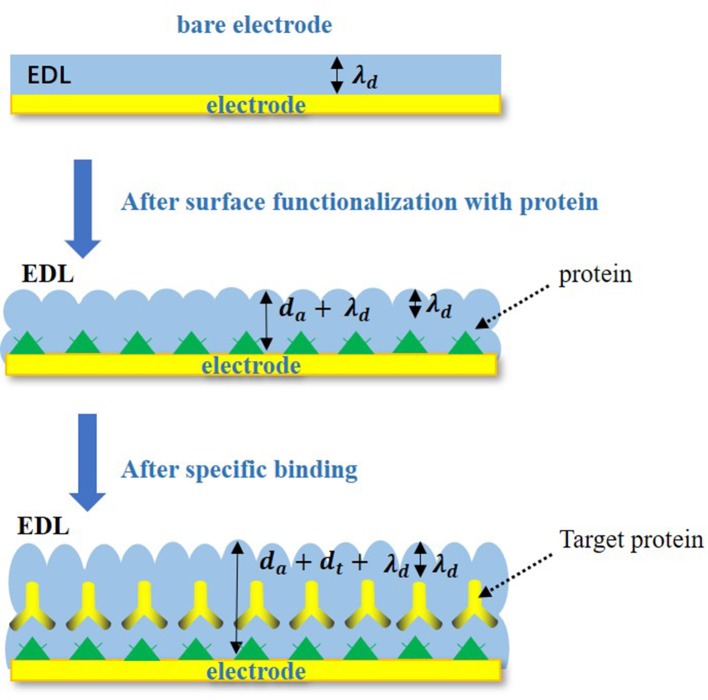
Specific binding changes on electrode surface.

In the Nyquist plots, we can calculate electrode resistance via the diameter of the semicircle. The larger the resistance, the larger the radius of the semicircle, which can directly reflect the changes in the electrode interface. In this work, when the solution was added to the electrode surface, the specific interaction between TBP protein and TATA-box, as well as the binding between TBP protein and BbMBF1 protein, will lead to the increase in the thickness of the electric double layers, resulting in the reduction of ***C***_***dl***_, as shown in Figure 3 (Helmholtz, 1879). According to the equation (1) shows that when the electric double-layer capacitance ***C***_***dl***_ decreases, the impedance ***Z***of the equivalent circuit will increase, the corresponding radius of Nyquist plots will also increase.

(1) $$\begin{array}{r} Z\;=\;R_{s}+\frac{1}{j\omega C_{dl}+\frac{1}{R_{ct}+\sigma w^{\frac{-1}{2}}\left(1-j\right)}} \end{array}$$

Where ***R***_***S***_ is the ohm internal resistance, ***R***_***ct***_ is the charge transfer resistance, ***C***_***dl***_ is the electric double-layers capacitance, **σ** is the diffused coefficient, **ω** is the angular frequency, ***j*** is the imaginary unit.

## Materials and Methods

### Materials and Reagents

*Beauveria bassiana* ARSEF2860 was purchased from RW Holley Center for Agriculture and Health, Ithaca, NY, USA. Expression carrier: pET-28-a (+) contains six histidine labels and was gained from Novagen (USA). T4DNA ligase was acquired from New England BioLabs. *Escherichia coli* Rosetta DE3 cells were purchased from Novagen (USA) and used for heterologous expression of proteins. *Escherichia coli* TOP10 were applied to vector transformation and plasmid amplification and purchased from Invitrogen (USA).

### Methods

#### Heterologous Expression and Purification of BbTBP and BbMBF1

Before heterogenous expression and purification of TBP and MBF1, expression carriers of TBP and MBF1 were constructed. In order to build pET-28a-BbTBP and pET-28a-BbTBP recombinant vector, we applied T4DNA ligase to connect the target gene to the vector. The recombinant carrier was transformed into *E. coli* TOP 10 for transformation and extraction, thus obtaining the recombinant plasmid. The recombinant plasmids pET-28a-BbTBP and pET-28a-BbMBF1 were transformed into *E. coli* Rosetta DE3 cells of protein-expressing. According to the characteristics of host bacteria, different inducers were added to induce protein expression. The expression levels of BbTBP and BbMBF1 proteins were optimized based on induction time and temperature. Optimal conditions for large-scale expression of BbTBP and BbMBF1 were selected. The protein was purified according to the label of recombinant protein, then concentrated and dialyzed to obtain BbTBP and BbMBF1 proteins. This process of expression and purification is described in more detail in article (Chi et al., 2015). Among them, the concentration induced by BbTBP heterologous expression of IPTG was 0.3 mM, the induction temperature was 18°C and induction time was 12 h. BbMBF1 induce expression of IPTG at a concentration of 0.5 mM, and the induction condition was 30°C for 4 h.

#### Methods

An e-corder impedance analyzer (EDAQ Instruments, USA) was applied in this work. The specific electrode is a three-electrode system, the test electrode is a graphene electrode, the reference electrode is a platinum wire, and the connection electrode is an Ag/AgCI (saturated KCI) electrode. The three electrodes were placed in the potassium iron hydride electrolyte to keep distance from each other to prevent short circuit. Before testing the sample, the electrode assembly was verified by cyclic voltammetry and impedance method. And the cyclic voltammetry has a working potential of 0 ~ 0.65 V and a sweep speed of 0.1 V/S.

**Figure 4:**
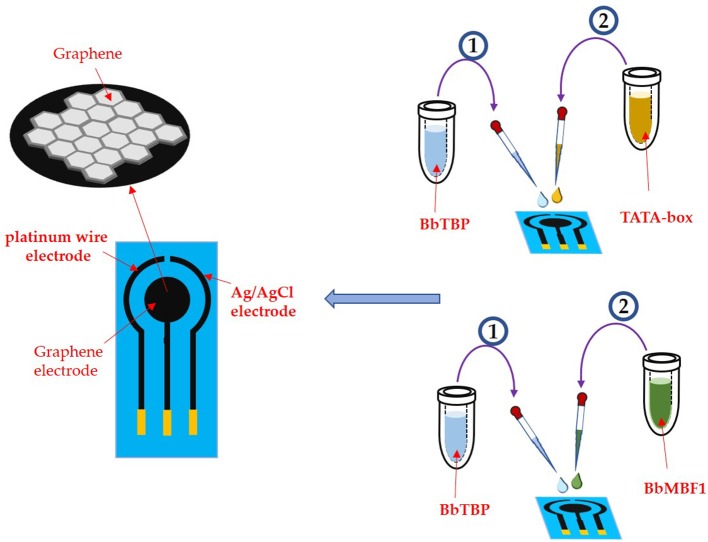
Experimental process.

As shown in Figure 4, 2 μl of the BbTBP protein sample contains a 6 × His label was added dropwise to the graphene electrode for functionalization. After the sample was dried, the graphene electrode surface was detected impedance value. Then add the BbMBF1 protein or the nucleic acid sequence TATA-box to be tested to the middle of the electrode, and after drying, measure the change in the surface impedance of the graphene electrode, and obtain the Nyquist plot. Using the impedance meter's own software ZMAN 2.2 to analyze the spectrum and obtain the impedance value of the electrode surface. In this work, BbTBP protein was firstly dropped onto the surface of graphene electrode and the changes of impedance on the electrode surface were measured. The Nyquist plot was acquired through measuring the current changes on the electrode surface via an electrochemical system, which could present the physical changes on the surface in a numerical way.

## Results and Discussions

### EIS Analysis of BbTBP and TATA-Box Probes

**Figure 5:**
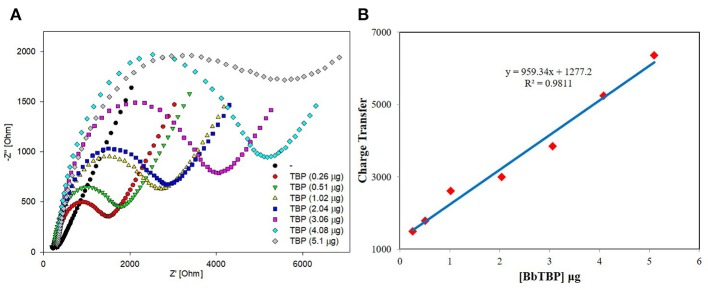
Sensitivity and linearity of different concentration of BbTBP protein detected with test electrode. **(A)** The EIS diagram of different concentration of BbTBP. **(B)** The impedance of the sensor increased proportionally to the concentration of BbTBP.

The sensitivity and linear relationship of BbTBP protein binding test electrode were tested firstly. Two microliter BbTBP protein (0.26, 0.51, 1.02, 2.04, 3.06, 4.08, and 5.1 μg) of different concentrations were dropped onto the electrode surface, and then the impedances of electrode surface were measured, respectively. In the Figure 5A, the radius of the semicircle increased with the increasing of concentration. Where the starting point of the semicircles were almost equal, the *R*_*s*_ resistance was almost unchanged. The increase of the radius represented the increase of the transfer resistance *R*_*ct*_, which reflected the strength of charge transfer at the interface. The stronger the charge transfer was, the larger the transfer resistance *R*_*ct*_ was, the smaller the interface capacitance *C*_*dl*_ was, and the impedance value on the corresponding electrode surface increased. This also indicated that with the increase of BbTBP protein concentration, more and more proteins adsorbed on the electrode surface, resulting in the increase in the thickness of the electric double layer and enhanced charge transfer, which increased *R*_*ct*_ and decreased *C*_*dl*_. And the impedance of the equivalent circuit was also increased. From Figure 5B, we could see that the impedance of the electrode surface increased with the change of BbTBP protein concentration, showing a certain linear relationship (*R*^2^ = 0.9811).

**Figure 6:**
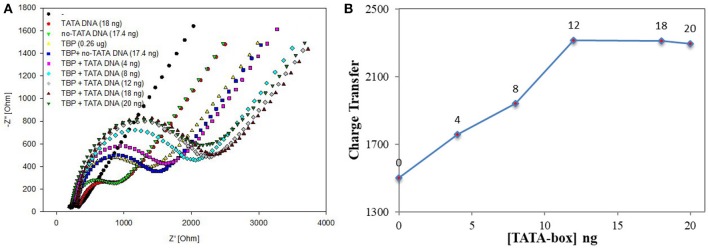
EIS analyzed BbTBP with different concentration of TATA-box 1. **(A)** The EIS diagram of BbTBP changed with the addition of TATA-box 1 at different concentrations. **(B)** The impedance of the sensor changed with different concentration of TATA-box 1.

According to the experiment and detection principle of the BbTBP protein binding test electrode, a low concentration of BbTBP protein (0.26 μg) was selected to detect its binding effect on TATA-box. Firstly, 2 μL, 0.26 μg of BbTBP protein was dropped onto the test electrode surface, and then 4, 8, 12, 18, and 20 ng TATA-box 1 nucleic acid sequences were sequentially added dropwise to the test electrode surface. The concentrations of TATA-box 1 is 80, 160, 240, 360, 400 nM. In order to evaluate the specificity of BbTBP and TATA-box, the concentrations 360 nM of TATA-box and no-TATA-box were kept as controls. At the concentration and same volume (2 μL), TATA-box 1 nucleic acid (24843Da) mass is 18 ng, and no-TATA-box nucleic acid (24073Da) mass is 17.4 ng. The nucleic sequences of tata-box and no-tata-box are shown in Table 1, the bold part is the TATA core sequence, and their masses are calculated by formula 2. Simultaneous detection of impedance changes on the test electrode surface. The results were shown in Figure 6A. It could be seen that the adsorptive behavior of TBP and controls were very different. But for control group, the Nyquist plots of the electrode surface without BbTBP protein were very similar. For TBP and TBP+no TATA DNA (360 nM), the Nyquist plots of the electrode surface were very similar. And for TBP and TBP+ TATA DNA (360 nM), the Nyquist plots of the electrode surface were very different. It could be seen from the figure that the radius of Nyquist plot of the electrode surface with BbTBP protein increased with the addition of TATA-box 1 at different concentrations, indicating that the impedance of the electrode surface increased after the addition of TATA-box 1, which further proved that BbTBP protein and TATA-box 1 had binding effect on the electrode surface. Figure 6B was the impedance corresponding to the Nyquist diagram.

(2) $$\begin{array}{r} \text{M}=\text{C}\times \text{V}\times {\text{M}}_{\text{r}} \end{array}$$

Where **M** is the mass, **C** is the concentration (mM), **V** is the volume (ml), **M**_**r**_ is the molecular weight (g/mol).

**Table 1:** The nucleic sequences of TATA-box and no-TATA-box.

**Name**	**Nucleic sequences**
TATA-box	CAGTAAAAGCTTGGTAGTATT**TATA**TCTTCTCTCTTTCAC
No TATA-box	CAGTAAAAGCTTGGTAGTATTTCTTCTCTCTTTCAC

### EIS Analysis of the Interaction Between BbTBP and BbMBF1

**Figure 7:**
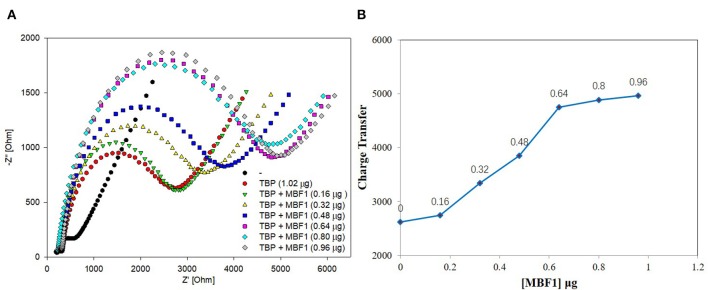
EIS analyzed BbTBP with different concentration of BbMBF1. **(A)** The EIS diagram of BbTBP changed with the addition of BbMBF1 at different concentrations. **(B)** The impedance of the sensor changed with different concentration of BbMBF1.

The results of BbTBP protein and TATA-box electrochemical experiments showed that the electrochemical impedance spectroscopy could be used for the interaction between protein and nucleic acid. Similarly, the electrochemical system was also used to try to detect the possibility of protein-protein interactions. Firstly, the His-tag of the BbMBF1 protein was removed, we used the thrombin cleavage site on the expression vector pET28a to remove the BbMBF1 protein. Then, the digested protein was dropped onto the surface of the test electrode, and the impedance of the electrode surface was detected to verify the effect of the enzyme digestion. The enzyme digestion conditions of His-tag were determined by comparing the impedance of the blank electrode. The results were shown in Figure 7. Compared with the blank electrode, the enzyme-digested BbMBF1 protein was less adsorbed on the electrode surface with the same impedance level as the negative control. This shows that the BbMBF1 protein His-tag was completely excised.

BbTBP containing His-tag of 1.02 μg was dropped onto the surface of the test electrode, and then the BbMBF1 protein containing His-tag of different concentrations were removed successively (0.16, 0.32, 0.48, 0.64, 0.8, and 0.96 μg). The impedance changes on the surface of the test electrode were detected by electrochemical system. As shown in Figure 7, it was obvious from Figure 7A that the semicircle radius increased with the increase of concentration of BbMBF1, which also reflected the binding effect between the BbTBP protein and BbMBF1 protein on the electrode surface. And with the increase of BbMBF1 protein concentration, the binding effect was enhanced, and binding was basically saturated at a certain concentration (0.64 μg). Figure 7B showed the impedance on the electrode surface with the increase of BbMBF1 protein concentration. It could be more intuitively seen from the figure that the changes of impedance on the electrode surface. This experiment showed that electrochemical impedance spectroscopy could also be used to detect protein-protein interactions.

**Figure 8:**
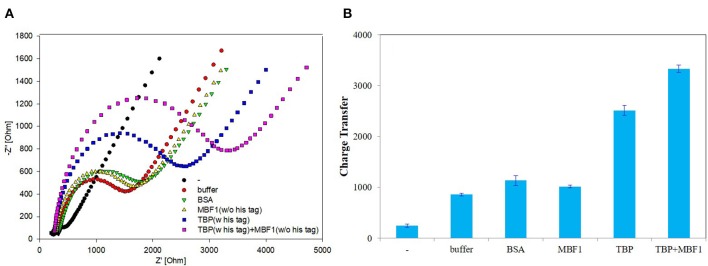
Control 1 of EIS analyzed BbTBP with different concentration of BbMBF1. **(A)** The EIS diagram of BSA, buffer, BbMBF1, and BbTBP + BbMBF1. **(B)** The impedance of the sensor changed with BSA, buffer, BbMBF1, and BbTBP + BbMBF1.

**Figure 9:**
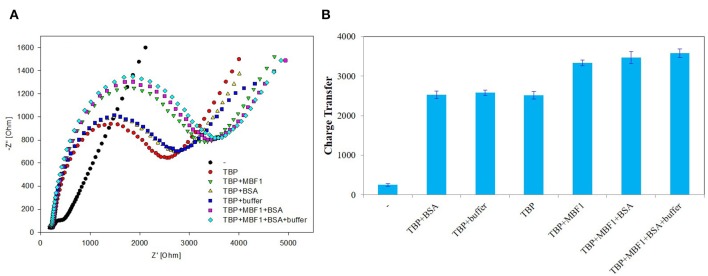
Control 2 of EIS analyzed BbTBP with different concentration of BbMBF1. **(A)** The EIS diagram of BbTBP, BbTBP + BbMBF1, BbTBP + BSA, BbTBP + buffer, BbTBP + BbMBF1 + BSA, and BbTBP + BbMBF1 + BSA + buffer. **(B)** The impedance of the sensor changed with BbTBP, BbTBP + BbMBF1, BbTBP + BSA, BbTBP + buffer, BbTBP + BbMBF1 + BSA, and BbTBP + BbMBF1 + BSA + buffer.

In order to further determine the accuracy of the experiment, a detailed control experiment was conducted, as was shown in Figures 8, 9. The 1.02 μg BbTBP protein and 0.48 μg BbMBF1 protein were selected for electrochemical detection of the interaction. BSA and buffer were added as controls. Figure 8 showed that the impedance on the surface of the BbMBF1 protein test electrode with only BSA, buffer and His-tag removed was only slightly higher than the blank electrode. After BbTBP was dropped, the impedance on the surface of the electrode was significantly higher. This indicated that the binding of BbTBP protein to the electrode was not disturbed by other factors. In addition, the effects of BbTBP protein and BbMBF1 protein were also unaffected by other factors.

## Discussion

To verify the interaction between BbTBP and TATA-box, this work tried a graphene biosensor based on electrochemical impedance spectroscopy. Firstly, BbTBP containing a histidine tag was combined with test electrode, which can specifically recognize TATA-box. By measuring the impedance changes of electrode surface to detect binding of BbTBP with TATA-box is effective and practical. Secondly, the interaction signal between BbTBP and BbMBF1 was acquired by the graphene-based electrochemical biosensors. The interaction between BbTBP and BbMBF1 was proved based on the results. Compared with gel migration experiment (Chi et al., 2015), DNase footprinting method and chromatin immunoprecipitation, the method has label-free, simple operation, and low-cost. In addition, the use of the target-specific probe DNA imparts extraordinarily high selectivity to the sensor, so it provides a simple detection method for the complex process of eukaryotic gene transcription regulation.

## Data Availability Statement

All datasets generated for this study are included in the article/supplementary material.

## Author Contributions

CS, ZP, and HL: methodology, formal analysis, investigation, data curation, writing—original draft. XL: conceptualization, validation, writing—review and editing, supervision, project administration, funding acquisition. MS, LJ, XX, and HJ: resources, writing—original draft.

### Conflict of Interest

The authors declare that the research was conducted in the absence of any commercial or financial relationships that could be construed as a potential conflict of interest.
